# Antibody-Mediated Osseous Regeneration for Bone Tissue Engineering in Canine Segmental Defects

**DOI:** 10.1155/2018/9508721

**Published:** 2018-02-28

**Authors:** A. Khojasteh, S. Hosseinpour, M. M. Dehghan, F. Mashhadiabbas, M. Rezai Rad, S. Ansari, S. Farzad Mohajeri, H. H. Zadeh

**Affiliations:** ^1^School of Dentistry, Shahid Beheshti University of Medical Sciences, Tehran, Iran; ^2^School of Advanced Technologies in Medicine, Shahid Beheshti University of Medical Sciences, Tehran, Iran; ^3^Students' Research Committee, School of Dentistry, Shahid Beheshti University of Medical Sciences, Tehran, Iran; ^4^Department of Surgery and Radiology, Faculty of Veterinary Medicine, University of Tehran, Tehran, Iran; ^5^Institute of Biomedical Research, University of Tehran, Tehran, Iran; ^6^Oral and Maxillofacial Pathology, School of Dentistry, Shahid Beheshti University of Medical Sciences, Tehran, Iran; ^7^Dental Research Center, Research Institute of Dental Sciences, School of Dentistry, Shahid Beheshti University of Medical Sciences, Tehran, Iran; ^8^University of California, Los Angeles, CA, USA; ^9^Laboratory for Immunoregulation and Tissue Engineering (LITE), Ostrow School of Dentistry, University of Southern California, Los Angeles, CA, USA

## Abstract

Among many applications of therapeutic monoclonal antibodies (mAbs), a unique approach for regenerative medicine has entailed antibody-mediated osseous regeneration (AMOR). In an effort to identify a clinically relevant model of craniofacial defect, the present study investigated the efficacy of mAb specific for bone morphogenetic protein- (BMP-) 2 to repair canine segmental mandibular continuity defect model. Accordingly, a 15 mm unilateral segmental defect was created in mandible and fixated with a titanium plate. Anorganic bovine bone mineral with 10% collagen (ABBM-C) was functionalized with 25 *μ*g/mL of either chimeric anti-BMP-2 mAb or isotype-matched mAb (negative control). Recombinant human (rh) BMP-2 served as positive control. Morphometric analyses were performed on computed tomography (CT) and histologic images. Bone densities within healed defect sites at 12 weeks after surgery were 1360.81 ± 10.52 Hounsfield Unit (HU), 1044.27 ± 141.16 HU, and 839.45 ± 179.41 HU, in sites with implanted anti-BMP-2 mAb, rhBMP-2, and isotype mAb groups, respectively. Osteoid bone formation in anti-BMP-2 mAb (42.99% ± 8.67) and rhBMP-2 (48.97% ± 2.96) groups was not significantly different but was higher (*p* < 0.05) than in sites with isotype control mAb (26.8% ± 5.35). In view of the long-term objective of translational application of AMOR in humans, the results of the present study demonstrated the feasibility of AMOR in a large clinically relevant animal model.

## 1. Introduction

Bone repair encompasses a cascade of biological processes that require progenitor cells, appropriate signaling molecules, and suitable scaffold [1]. Administration of bioactive molecules such as growth factors [2, 3] and cytokines [4] can mediate bone regeneration by driving stem cell into osteogenic differentiation. Growth factor therapy has been utilized for bone tissue engineering with promising results by application of osteoinductive exogenous growth factors including BMPs, fibroblast growth factor (FGF), transforming growth factor-beta (TGF-*β*), platelet-derived growth factor (PDGF), insulin-like growth factor (IGF), and vascular growth factor (VEGF) [5–9]. BMP family as a member of TGF-*β* superfamily [10] consists of over 20 identified members, not all of which have osteoinductive function [11]. BMPs initiate BMP/TGF-*β* signaling pathways that culminate in osteogenesis [12, 13]. Recombinant human (rh) BMP-2 and BMP-7 [14] have been approved for specific indications of skeletal repair by US Food and Drug Administration (FDA) [15]. Application of BMPs showed bone formation initiation [16], stem cells differentiation and migration [16, 17], and promotion of bone volume [18, 19], area [20], calcium content [21], and mechanical strength [22]. BMP-2 plays a significant role in BMP and Wnt signaling and is essential for inherent capacity of bone healing [23–25]. In addition, application of exogenous rhBMP-2 has been demonstrated to initiate the bone regeneration cascade through the BMP/TGF-*β* pathway [26, 27].

Notwithstanding the positive osteogenic effects of rhBMP-2 clinical administration, such strategy has a number of drawbacks that limit this mode of therapy as the definitive solution. Specifically, recombinant growth factors have lower activity than endogenous counterparts, unsustainable concentration over time, and short* in vivo *half-life [28–32], necessitating the clinical dosage, which is several orders of magnitude greater than the physiologic dose. The extremely high dose has been attributed to growing concerns about biological complications, such as increased malignancy risk [33] and potentially life-threatening edema. A practical disadvantage of administrating high dosage of protein required achieving the desired clinical response; the cost is proportionally high [34].

Hence, alternative therapeutic approaches such as gene therapy [35], protein therapy [36–38], and antibody therapy [39–41] have been proposed to overcome these limitations. Moreover, antibody-mediated osseous regeneration (AMOR) technique has been documented as an alternative to the application of exogenous rhBMP-2. This principle is based on an approach to use specific monoclonal antibodies (mAb) such as anti-BMP-2 to capture endogenous BMPs to mediate bone regeneration [42]. Previous studies indicated AMOR as a promising alternative in bone tissue engineering* in vitro *and* in vivo *[39, 42–48]. In critical sized calvarial defects in rat and rabbit models, complete repair has been observed after 6–8 weeks [39, 42–48] and similar defect among rabbits showed significant increased amount of new bone formation after 45 days [45]. It is essential to investigate the influences in larger models due to mimicking the actual clinical conditions [49]. In the present study, we created segmental mandibular defects in canine models.

The main objective of this study was to investigate the feasibility of applying chimeric anti-BMP-2 mAbs for AMOR used in segmental mandibular defects of canine models for bone regeneration.

## 2. Materials and Methods

### 2.1. Reagents

Anorganic bovine bone mineral with 10% collagen (ABBM-C; Bio-Oss-Collagen, Geistlich Pharma, Wolhusen, Switzerland) was used as the scaffold in AMOR. ABBM has interconnected macropores to allow for neovascularization and bone cell infiltration and micropores to allow for fluid exchange, as well as nanostructure to help with osteogenesis [50]. Chimeric anti-BMP-2 mAb recently developed in our laboratory [47] was used in this study. The negative control consisted of isotype-matched mAb (Iso, anti-rabbit IgG2a mAb, Biovision, Mountain View, CA) with no specific affinity to BMP-2. Anti-BMP-2 mAb and its isotype-matched control mAb were diluted with phosphate-buffered saline (PBS) at 25 *μ*g/mL concentration. Recombinant human BMP-2 (Infuse, Medtronic, Inc., Memphis, TN, USA) was used at 20 *μ*g/ml as positive control. Scaffolds were incubated with 250 *μ*l of diluted anti-BMP-2 mAb (total of 6.25 *μ*g), isotype-matched mAb (total of 6.25 *μ*g), or rhBMP-2 (total of 5.0 *μ*g), as previously described [42].

### 2.2. Animals

Nine male mongrel dogs with average weight of 14–22 kg, aged 1-2 years, were used in this study. This experiment was approved by Institutional Animal Care and Use Committee (IACUC) of the Shahid Beheshti University of Medical Sciences and conformed to standards of the Association for Assessment and Accreditation of Laboratory Animal Care. The animals were kept for 2 weeks to become acclimatized to housing and diet. Animals were vaccinated and treated by antifungal drugs by staff veterinarian. During the study, animal well-being was monitored by examining activity, general appearance, and weight. They were fed soft food (Friskies, Purina, Marne La Vallee, France).

### 2.3. Surgical Procedure

**Figure 1:**
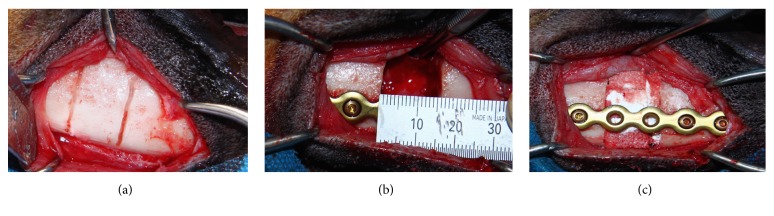
Intraoperative clinical images of the steps in segmental osteotomy and repair. Extraoral surgical approach to expose the mandible and depth grooving to mark the exact defect size (a). Fixation of mandibular segments with titanium reconstruction plate (b). Segmental mandibulectomy (15 mm) was performed and a titanium plate was used to rigidly fixate the two segments together. The resected mandibular segmental defect was implanted with anorganic bovine bone mineral collagen, functionalized with anti-BMP-2 mAb or isotype-matched control mAb (c).

Systemic (ketamine hydrochloride, Ketavet, 5 mg/kg body weight) and local anesthesia (2% lidocaine with 1 : 80,000 epinephrine) were administered. Mandibular body was exposed by an extraoral submandibular incision (Figure 1(a)). Prior to creating defects, one 9-hole titanium reconstruction plate was adapted to the inferior border of mandibular buccal side in order to keep mandible in appropriate position. The plate was secured via titanium screws. To create a segmental defect of 15 mm width in the mandibular fourth premolar tooth (PM4) on right side of each mandible [51] plate and screws were removed temporarily. After the resection, they were repositioned (Figure 1(b)). The surgical region was copiously irrigated with sterile saline solution. The animals were randomly divided into three groups of 3 dogs each: (A) Bio-Oss collagen + anti-BMP-2 mAb (25 *μ*g/mL), (B) Bio-Oss collagen + rhBMP-2 (20 *μ*g/mL), and (C) Bio-Oss collagen + isotype-matched mAb (25 *μ*g/mL). After placement of scaffolds (Figure 1(c)), the periosteum and skin were approximated and sutured in two layers by resorbable suture (Vicryl 3.0; Ethicon GmbH & Co., KG, Norderstedt, Germany). Thirty minutes before surgery, each dog received a single intramuscular injection of antibiotic (ampicillin 100 mg/kg) and analgesic (morphine 2 mg/kg). The animals were humanely euthanized at 12 weeks postoperatively with vital perfusion-fixation technique through carotid artery [52].

### 2.4. Radiographic Analysis

Computed tomography (CT) image acquisition was conducted immediately postoperatively for baseline and repeated after 6 and 12 weeks. Dogs were scanned by a high-resolution CT imaging machine (Siemens Somatom Spirit; Berlin, Germany). The effective voxel size was 500 *μ*m and three-dimensional reconstruction of the images was performed using a “bone mask” in Vitrea Core (v.6.3.2089.106; VITAL, Minnetonka, MN). Bone density was recorded in Hounsfield Unit (HU) within the regions of interest defined within the confines of the defects.

### 2.5. Histological and Histomorphometric Examination

Mandibular specimens were fixed with 10% formalin (Richard-Allan Scientific, Kalamazoo, MI) for 24 hours at 24°C. After dehydration in ascending concentration of ethanol and clearance in xylene (Sigma-Aldrich), they were embedded in paraffin. The role of sectioning is demonstrated in schematic diagram (Figure 4(a)). Each specimen was divided into three equal sections in which the central part was cut into five-micrometer sections with width parallel to frontal plane. In order to achieve delicate view of histological junction between new bone formed and native bone, distal and proximal parts were cut into five-micrometer sections with width parallel to transverse plane. The sections were stained with hematoxylin and eosin (H&E). The most central sections were analyzed by quantitative histomorphographic assessment of stained slides by a PC-based image analysis system (Image-Pro Plus, Media Cybernetic, Silver Spring, MD). One calibrated examiner determined new bone formation, residual body, soft tissue, and inflammatory elements by polarized light microscopy (Olympus, SZX 9, Tokyo, Japan).

### 2.6. Statistical Analysis of Data

In order to determine statistically significant differences between the experimental groups, a nonparametric analysis of variance (Kruskal-Wallis) was used. One-way analysis of variance (ANOVA) was used for the comparison of means in histomorphometric quantitation. General linear model of repeated measures was used for comparison of mineralized area and bone density within the groups. After all, Tukey's test was utilized as the post hoc test at a significance level of 0.05 (SPSS 16, SPSS Inc., Chicago, IL).

## 3. Results

### 3.1. Clinical Outcomes

Gross examination of reconstructed defects revealed the presence of a pale yellow fibrous tissue surrounding the previous defect sites in all specimens. The position of screws and plates were stable in all specimens at experimental end-point. The tissues formed within sites grafted with anti-BMP-2 mAb and rhBMP-2 groups appeared to be uniformly solid, compared to control sites which were soft and rubbery.

### 3.2. Radiographic Analysis

**Figure 2:**
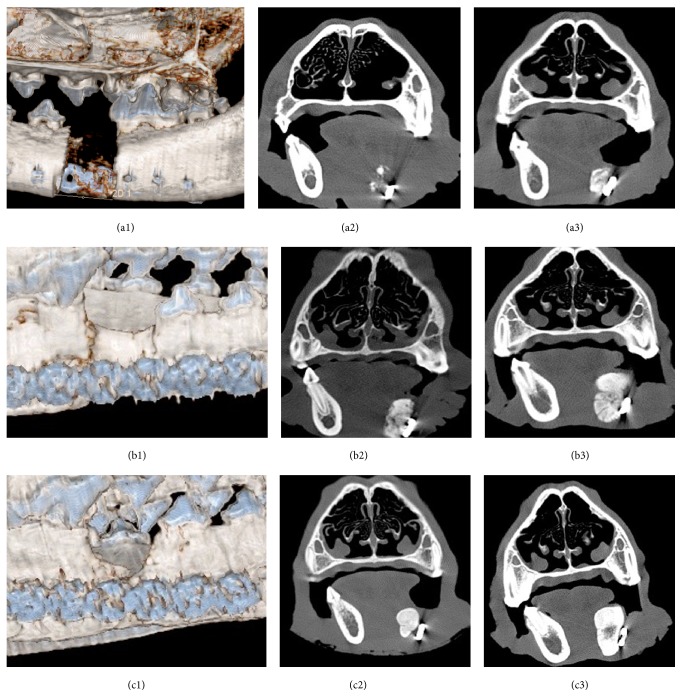
CT scan images of the bone formation within canine segmental defects. Three-dimensional images of the defects after 6 weeks in isotype mAb (a1), rhBMP-2 (b1), and anti-BMP-2 (c1) groups. Coronal sections of the defect's center slide were shown at 6th week in isotype mAb (a2), rhBMP-2 (b2), and anti-BMP-2 mAb (c2) groups. Same sections at 12th week in isotype mAb (a3), rhBMP-2 (b3), and anti-BMP-2 (c3) groups.

Representative CT imaging data are shown in Figure 2, illustrating qualitative differences among the groups. These 3D reconstructed CT images provide evidence for less defect fill within isotype control mAb group (a1), as compared with anti-BMP-2 mAb and rhBMP-2 groups. The regenerated tissues in site treated with rhBMP-2 appeared to be separated from host bone by a demarcation line (b1). In contrast, the defects treated with anti-BMP-2 showed smooth regeneration without any demarcation line between old and new bone (c1). Comparison of coronal sections (a2, a3, b2, b3, c2, and c3) showed clear differences among the 3 treatment groups. Anti-BMP-2 group showed a homogenous regenerated bone differentiated into medullary and cortical regions, resembling native bone. Although sites treated with rhBMP-2 showed evidence of radiopaque tissue formation, the bone did not exhibit the same level of organization of cortical/cancellous seen in normal host bone. The bone formation in isotype mAb group was attenuated with small noncontiguous islands in some specimens.

**Figure 3:**
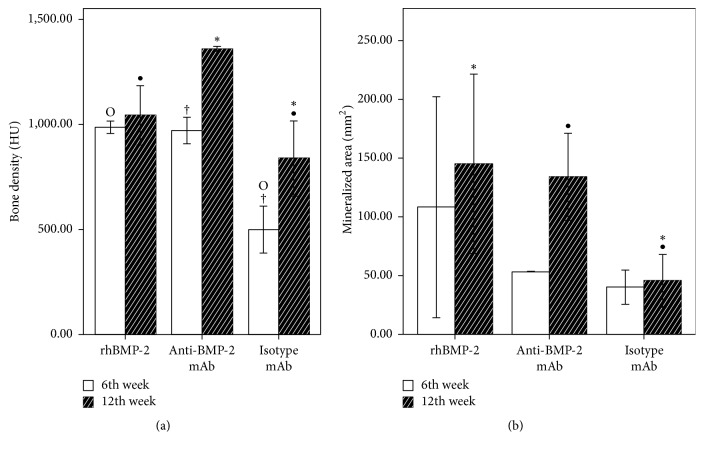
Quantitative analysis of calcified tissues in the defect sites by CT scan imaging. (a) Comparison of bone density (Hounsfield Unit) of isotype mAb, rhBMP-2, and anti-BMP-2 mAb at 6th and 12th weeks. (b) Comparison of mineralized area (mm^2^) of isotype mAb, rhBMP-2, and anti-BMP-2 mAb at 6th and 12th weeks. Means and standard deviations were calculated in each group and statistical significance was assessed by general linear models for repeated measures and Tukey's test as the post hoc test (^*∗*,•,O,†^*p* < 0.05; all other comparisons in each time point were not significant).

Quantitative morphometry was conducted on the CT images and results are shown in Figure 3. These morphometric data at 6 weeks demonstrated increased mineralized bone surface only in positive control rhBMP-2 (108.38 + 93.8) groups, compared with experimental anti-BMP-2 mAb (43.22 ± 17.88) and isotype-matched negative control group (40.52 ± 14.51). By 12 weeks, mandibular defects treated with anti-BMP-2 mAb (133.92 ± 37.02 mm^2^) or rhBMP-2 (145.16 ± 76.17 mm^2^) exhibited increased mineralized bone area, compared with isotype-matched negative control group (46.01 ± 22.25 mm^2^). The comparison of mean mineralized area showed significant difference between experimental and positive control groups compared with negative control at both time points (*p* < 0.05). However, there was no statistical difference between anti-BMP-2 mAb and rhBMP-2 groups (*p* > 0.05) (Figure 2).

Bone densities in anti-BMP-2 mAb, rhBMP-2, and isotype mAb (control) groups were 1360.81 ± 10.52 HU, 1044.27 ± 141.16 HU, and 839.45 ± 179.41 HU at 12th week (Figure 2). The statistical analysis indicated significantly greater bone density in anti-BMP-2 mAb and rhBMP-2 compared with isotype mAb (*p* < 0.05). However, bone densities were not statistically significantly different in anti-BMP-2 mAb compared to rhBMP-2 treated groups (*p* > 0.05) (Figure 3).

### 3.3. Histological and Histomorphometric Analysis

**Figure 4:**
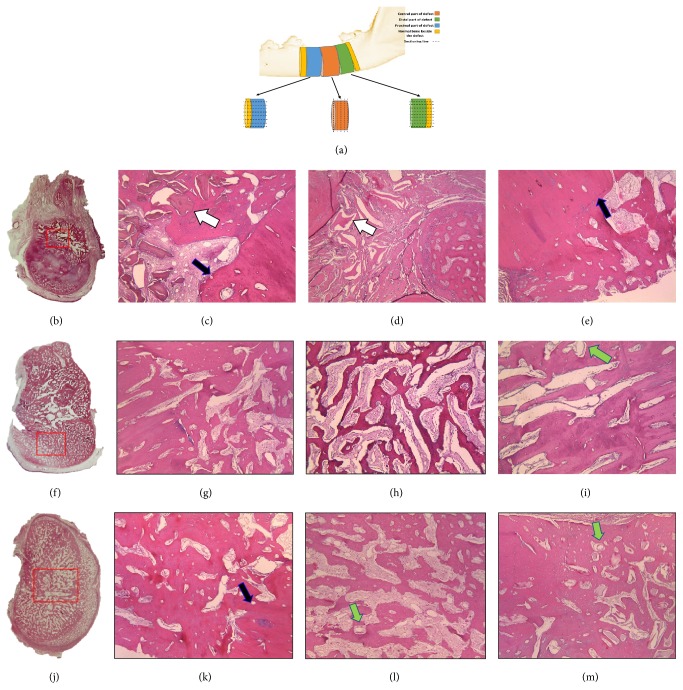
Histological examination of specimens at 12 weeks postoperatively. (a) Schematic diagram of the segmental defects, illustrating the location of sections taken for histological analysis. The proximal and distal segments were sectioned horizontally and the cross section of the central segment was taken (b, f, j). (b–m) Representative histomicrograms stained with H&E (40x). (b–e) Isotype-matched mAb, (f–i) rhBMP-2, and (j–m) anti-BMP-2 mAb used to functionalize ABBM-C scaffold. The blue arrows show the junction of newly formed bone and the neighboring host bone. The white arrows show the residual scaffold biomaterials, which were surrounded by connective tissue in control specimens treated with isotype-matched control mAb, while in experimental and positive control specimens the residual scaffold was not easily discernable. Abundant endothelial-lined blood vessels (green arrows) were noted in anti-BMP-2 mAb and rhBMP-2 treated sites.

Histological analysis of treated segmental osteotomy defects was carried out and results are shown in Figure 4. Osteotomy defects were divided into proximal, central, and distal portions (Figure 4(a)). Cross sections of the central portion were made to assess de novo bone formation (Figures 4(b), 4(f), and 4(j)). The proximal and distal portions were sectioned horizontally to allow assessment of osteogenesis at the junction of host bone and defect sites. Results showed significant de novo bone formation within central portions of sites treated with scaffolds functionalized with anti-BMP-2 mAb (Figure 4(j)) or rhBMP-2 (Figure 4(f)), compared to isotype-matched mAb (Figure 4(b)). The newly formed bone within defects were reconstructed with anti-BMP-2 mAb or rhBMP-2 bridged across the defects continuously from distal to proximal ends. The tissue microstructure showed new osteoid bone formation within defects treated with anti-BMP-2 mAb with mostly lamellar organization, containing osteocytes within lacunae and rimmed with osteoblasts. Bone formation within isotype control specimens was limited to regions immediately adjacent to defect edges, where new bone had formed by apposition on old host bone. Only minimal inflammatory infiltrate was observed, which was mainly superficial in all 3 groups.

**Figure 5:**
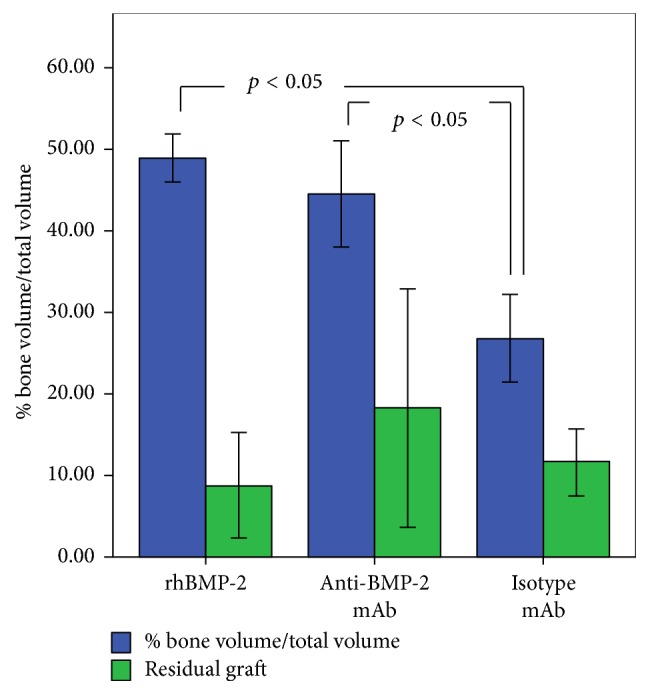
Quantitative histomorphometric analysis of bone specimens for bone volume/total volume (%) and residual graft (%). Means and standard deviations of each group were calculated and statistical significance was assessed by ANOVA and Tukey's post hoc test.

Histomorphometric evaluations revealed significantly more osteoid bone formation in anti-BMP-2 mAb (42.99%  ± 8.67) and rhBMP-2 (48.97%  ± 2.96) groups, compared with isotype mAb (26.8%  ± 5.35) (*p* < 0.05) (Figure 5). Difference of residual scaffold material was not statistically significant among the three groups.

## 4. Discussion

Since the introduction of the concept of AMOR, the main focus of our research team has been to pursue translational applications of scaffolds functionalized with anti-BMP-2 mAb for repair of major skeletal defects. We have pursued application of anti-BMP-2 in progressively larger animal models in clinically relevant models of skeletal defects. The current study sought to investigate the hypothesis that anti-BMP-2 can mediate repair of continuity mandibular defects. To that end, continuity mandibular defects were created in canine model and repaired with ABBM/collagen scaffold functionalized with chimeric anti-BMP-2 mAb, isotype-matched control mAb (negative control), or rhBMP-2 (positive control). AMOR group mediated 15.19% more bone formation than isotype mAb control group (*p* < 0.05). AMOR and rhBMP-2 groups did not yield significant differences in bone repair (*p* > 0.05). These results are consistent with other applications of AMOR and rhBMP-2 in repair of critical size defects [39, 43, 44, 47, 48]. These results are remarkable, when compared with the degree of bone formation achieved after autogenous bone augmentation in mandibular segmental canine model. Sverzut et al. demonstrated 43.35%  ± 2.87 in combination of microporous membrane and iliac graft and 60.98%  ± 2.38 in iliac graft alone for reconstruction of 10 mm segmental defects [53]. Hussein et al. conducted a study to compare the impact of rhBMP-2 delivery for reconstructing 35 mm long segmental mandibular canine defects after 12 weeks. They showed superior results of bone healing and in agreement with our results the percentage of new bone formation was 52.4%  ± 5.6 [54]. However, they reported more complications during their follow-up period.

The presence of bone formation in central sections was the evidence of de novo bone formation. Interestingly, the distal parts showed more bone formation than proximal parts in all groups. The tissue filling negative control defects were mostly fibrous in nature and constricted in dimensions. Most of the sparsely formed new bone was detected in the center and inferior part of the defect close to the overlying periosteum. The specimens containing rhBMP-2 and anti-BMP-2 revealed very well-formed bony trabeculae, throughout the defect with an active cuboidal osteoblast in the rim. There was direct contact between the original and new bone at the distal and proximal sections of the defect in the experimental group. The morphology of the bone forming within defects in the AMOR group mimicked native bone more closely than rhBMp-2 group. The trabecular pattern observed in the rhBMP-2 group was predominantly concentrated in the periphery, while in the AMOR group the trabecular pattern was more homogeneously distributed throughout the newly regenerated tissue.

Application of anti-BMP-2 mAb has a number of advantages over administration of exogenous rhBMP-2; for example, (1) there is higher biological activity of endogenous BMP-2 (nearly 10-fold higher) compared with recombinant counterpart [55], hence requiring lower concentrations of BMPs; (2) anti-BMP-2 captures multiple osteogenic endogenous growth factors with which it cross-reacts, including BMP-2, BMP-4, and BMP-7 [47, 48]; and (3) the BMPs captured can be sustained for several weeks in local sites [46–48]. Bone healing can synergistically promote by BMP-4 [56] and BMP-7 [32] in combination with BMP-2 [57]. The efficient dose of rhBMP-2 required for clinical influence is higher than physiological amount [58] and also rhBMP-2 has a short half-life in the body compared to antibodies, that is, 7 min to 2 days for rhBMP2 and 30 to 100 days for antibodies [47].

Radiographic analysis represented higher bone density in anti-BMP-2 mAb group (1360.81 ± 10.52 HU) compared to rhBMP-2 (1044.27 ± 141.16 HU) that is in accordance with our histological results indicating better quality of bone formation in anti-BMP-2 mAb group. Arzi et al. [59] demonstrated regenerated bone in rhBMP-2 group accomplished 46 to 54% of the density of the normal contralateral mandibular bone density after 12 weeks. However, our results showed comparable bone density to normal bone not only in the rhBMP-2 group but also in the anti-BMP-2 mAb group.

Therapeutic antibodies open a new window in regenerative medicine. Dozens of therapeutic monoclonal immunoglobulins are currently in clinical use or under development for the treatment of cancers [60, 61], osteoporosis [62], and immune disorders [60] to bone defects [39, 42–48, 63]. Recent studies showed bone healing enhancement in various bony fractures and defects within animal models [39–48, 63–71]. However, there are many adverse reactions associated with mAb therapy that encompass cytokine release syndrome, serum sickness, and tumor lysis syndrome [72]. Systemic usage of mAbs can vary from local immune reaction in the site of injection to severe acute anaphylactic shock [72]. In the current study, we applied anti-BMP-2 mAb locally in which attentive histological analysis failed to find any significant inflammation or other adverse reactions within treated defect sites. In comparison to systemic utilization of Sclerostin mAb for bone healing (25 mg/kg/dose twice weekly for at least 4 weeks) [41, 66], we used noticeably lower concentration of therapeutic mAbs (6.25 *μ*g of total used in each defect). Therefore, application of therapeutic monoclonal antibodies for bone tissue engineering has potentially significant benefits for repair of major skeletal defects. Further investigation is merited prior to clinical application of AMOR.

## 5. Conclusion

The results of present study made a number of important observations, including (1) evidence of de novo bone formation in a large clinically relevant skeletal defect model, (2) demonstration of the efficacy of anti-BMP-2 for the first time in the canine model, and (3) lack of adverse reaction to the local application of anti-BMP-2 mAb for repair of a large bony defect. The application of anti-BMP-2 mAb in concert with a commonly used scaffold of anorganic bovine bone mineral and collagen yielded successful de novo bone formation to an extent, which was equivalent to that of FDA-approved rhBMP-2, with no evidence of adverse reaction.
